# Perioperative management of a patient with a giant ovarian tumor: A case report

**DOI:** 10.1097/MD.0000000000022625

**Published:** 2020-10-09

**Authors:** Shengnan Cai, Rui Dai, Junqiao Mi, Shiduan Wang, Yan Jiang

**Affiliations:** Department of Anesthesiology, the Affiliated Hospital of Qingdao University, Qingdao, Shandong, China.

**Keywords:** anesthesia, complication, giant ovarian tumor, management, multidisciplinary approach

## Abstract

**Rationale:**

Giant ovarian tumors are very rare. Patients with large ovarian tumors appear similar to pregnant women and morbidly obese patients. The management of such patients is associated with significant mortality. Therefore, additional clinical research is essential to understanding the perioperative complications of this disease.

**Patient concerns:**

We report the perioperative management of a patient with a giant ovarian tumor that contained 23 L of fluid who underwent tumor resection. Given the infrequency of these giant ovarian tumors, a detailed anesthetic plan and postoperative respiratory support strategy were tailored to address the patient's hemodynamic and respiratory risks, as well as to minimize potential complications, including supine hypotensive syndrome, re-expansion pulmonary edema, and postoperative intestinal ileus. To prevent supine hypotensive syndrome, the patient used a mild left-sided position (10∼20°) after admission until the tumor was removed. In order to prevent re-expansion pulmonary edema (RPE), the intraoperative ventilator mode was set to pressure-controlled ventilation (PCV), with the addition of 8 cmH_2_O positive end-expiratory pressure (PEEP). The airway pressure was lower while maintaining a certain tidal volume. In the ICU, in the ventilator mode, we use pressure support ventilation as well as PEEP and adjust it according to the patient's spontaneous breathing situation and blood gas analysis to prepare for further detach from the respirator and extubation. And we prevent the occurrence of postoperative intestinal ileus by placing the abdominal binder after the operation.

**Diagnosis:**

Mucinous cystadenoma of the left ovary.

**Interventions:**

The patient underwent exploratory laparotomy with debulking of the left ovarian mass, transabdominal hysterectomy with bilateral salpingo-oophorectomy, complete omentectomy with appendectomy, and pelvic lymphadenectomy.

**Outcomes:**

After surgery, the patient experienced intestinal distention. Up to now, the patient has recovered well.

**Lessons:**

A multidisciplinary approach is essential. Knowing the possibility of complications and choices for management can lead to favorable outcomes in such rare cases. This case reminds us that postoperative complications such as postoperative intestinal ileus may be fatal.

## 1 Introduction

As is typical of all primary mucinous tumors, cystadenomas are characteristically unilateral. Only approximately 5% are bilateral. Cystadenomas are generally large tumors that can occasionally reach massive proportions.^[1]^ Such large ovarian tumors are rarely seen in current medical practice, as most of the cases are diagnosed early during routine gynecological examinations or through incidental findings on ultrasound examinations of the abdomen. Large intra-abdominal tumors are seen infrequently in modern surgical practice and are often regarded as a challenge. The gynecologist and anesthetist should be familiar with the alterations in physiology that large tumors will produce and should have adequate facilities available to manage any problems that may be occur during the perioperative period. We reported the perioperative management of a relatively rare case of a large ovarian tumor. We successfully resected the tumor and successfully treated the complications of postoperative intestinal distention.

## 2 Consent

The patient provided informed consent to collect data and images for publication. Ethical approval was not necessary in case of case report publication.

### 2.1 Case presentation

A 66-year-old female patient had no obvious cause of abdominal distension that started more than 3 years ago, and she experienced an increase in abdominal circumference (not controlled by diet); however, the patient was not admitted to the hospital for examination and treatment.

Recently, the patient felt that the bloating was aggravated, she could not be in a supine position, and she had difficulty breathing after physical activity. The patient chose to be admitted to the hospital.

The patient was usually healthy and had no other diseases. She was 161 cm tall, weighed 68 kg, and had an abdominal girth of 110 cm. She was G3P1A2. The admission test showed a cancer antigen (CA)125 level of 75.13 U/mL. Her hematological investigations were within normal limits, albumin 30 g/L and hemoglobin 109 g/L in the blood. Her liver, renal functions were also in normal values

**Figure 1:**
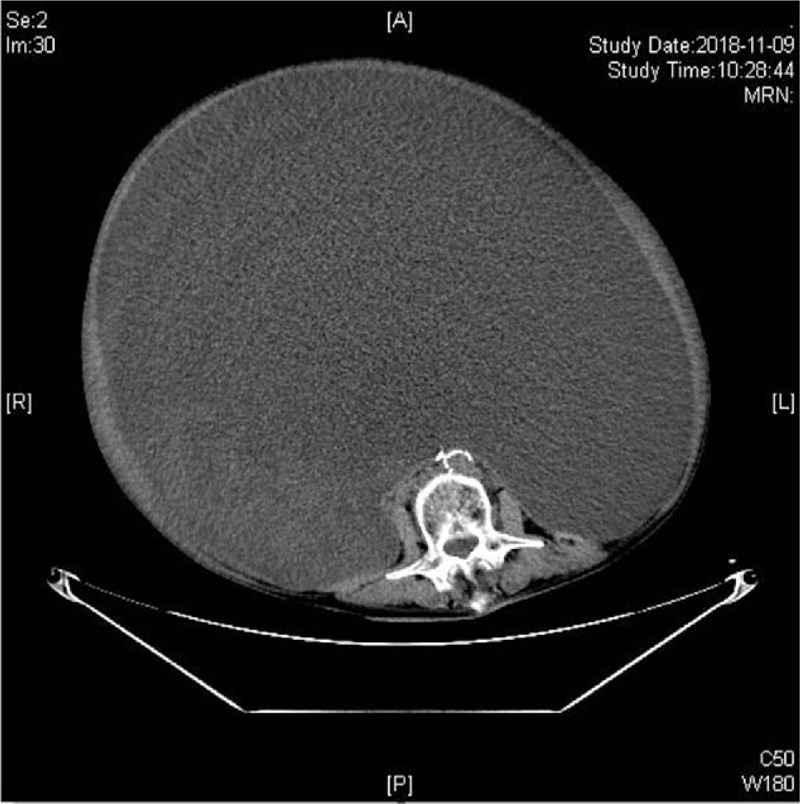
Preoperative abdominal CT shows a giant cystic tumor.

The patient's urinary system ultrasound showed right hydronephrosis and right ureteral dilatation. A chest computerized tomography (CT) scan showed coronary calcification and left pleural effusion. The abdominal enhanced CT showed a bulky cystic mass that arose from the left ovary and occupied the whole abdominal and pelvic cavity (Fig. 1). Her electrocardiogram showed sinus tachycardia. An examination of her cardiovascular system showed no abnormalities. Physical examination revealed mild edema of the lower extremity, and the patient reported that the urine volume decreased.

To prevent potential complications during the perioperative period, the gynecology team organized preoperative multidisciplinary teamwork (MDT) based on the patient's condition. The preoperative MDT included members of the gynecology, oncology, medical imaging, anesthesiology, urology, pathology, intensive care, and gastrointestinal surgery departments and operating room care units who convened before surgery to discuss the tumor pathology type, surgical technique, anesthesia protocol, physiologic considerations, assessment of other organ functions, postoperative recovery, and logistical support issues.

Considering the specific physical condition of the patient, we performed a full preoperative evaluation. Preoperative pulmonary function tests indicated restrictive impairment. On the basis of the assessment of the patient's condition and physical condition, we determined that the patient's was American Society of Anaesthesiologists (ASA) grade III.

Before surgery, 6 units of blood were ordered, and the availability of fresh frozen plasma and platelets was confirmed. The patient fasted from the day before surgery. Preoperative intramuscular injection of midazolam was treated as premedication in order to relieve the patient's anxiety. From admission to removing giant tumor, the patient received treatment in the mild left lateral position to avoid inferior vena cava compression (10∼20°). As prophylaxis against thromboembolism, the patient wore elastic support stockings before surgery.

Standard monitors were applied in the operating room. Noninvasive blood pressure (NIBP), bispectral index (BIS), and temperature monitors were connected. The baseline vital signs showed the following: BP: 128/88 mm Hg, heart rate (HR): 114 beats/min, breathing rate: 26 times/min, and body temperature: 36.3°C. A catheter was placed in the left radial artery and the right internal jugular vein under ultrasound guidance to monitor arterial blood pressure (ABP) and central venous pressure (CVP). We successfully performed epidural catheterization in the L_1-2_ segment of the lumbar spine for postoperative epidural analgesia.

Because heat loss was anticipated from the large surface area of the operation site, we used a heater to keep the patient warm. All intravenous fluids were warmed before administration. The operating theater temperature was maintained at 24°C, and the operating room doors were kept closed. The oropharyngeal temperature was measured continuously.

The patient with a large abdominal tumor could be treated as a full stomach. First, a dexmedetomidine infusion was administered for sedation at 1 μg/kg for 10 minutes along with 3 L/min O_2_ via a face mask, which was then followed by a rapid induction through a vein: etomidate: 20 mg, sufentanil: 15 μg, and rocuronium 42 mg with cricoid pressure. A tracheal tube with an internal diameter (ID) of 7.0 mm was successfully inserted under the guidance of a video laryngoscope. Intraoperative anesthesia was maintained by a continuous infusion of propofol according to the BIS target; sufentanil was added intermittently as needed to obtain a clinically adequate depth of anesthesia, and cisatracurium was continuously administered at 0.08 mg/kg/h. These drugs were chosen for their minimal and predictable effects on cardiovascular stability and their suitability for use during postoperative ventilation. We injected the patient with 50 mg flurbiprofen and 40 mg antiemetic ondansetron before the surgery was over.

After inducing anesthesia, the inspired oxygen fraction (FIO_2_) was 0.5. The tidal volume (TV) was set at 7 mL/kg predicted body weight with an initial respiratory frequency of 12 breaths/min and positive end-expiratory pressure (PEEP) of 5 cmH_2_O. The respiratory rate was adjusted to maintain an end-tidal carbon dioxide (P_ET_CO_2_) of 35 to 45 mm Hg. The inspiratory/expiratory ratio was 1:2, and the patient's airway pressure was 23 cmH_2_O. Once an abdominal midline incision was made by the surgeon, the TV was reduced gradually by anesthesiologist to preventing pulmonary edema. During the operation, the patient's airway pressure further decreased and stabilized as the tumor fluid was expelled.

The patient's pulse and BP remained stable until the peritoneal cavity was opened and the was cyst punctured. After anesthesia was induced, a catheter was inserted into the tumor from the lower abdomen to discharge the ovarian contents, and the suction speed was set to 1 L/min. In total, 23 L liquid was extracted over 30 minutes. During fluid extraction, the patient's ABP gradually decreased.

To maintain a stable patent's cardiac output and stroke volume, dopamine was started at 15 μg/kg/min and was then tapered to 5 μg/kg/min after the BP had stabilized. During this period, ephedrine and norepinephrine was administered as appropriate.

**Figure 2:**
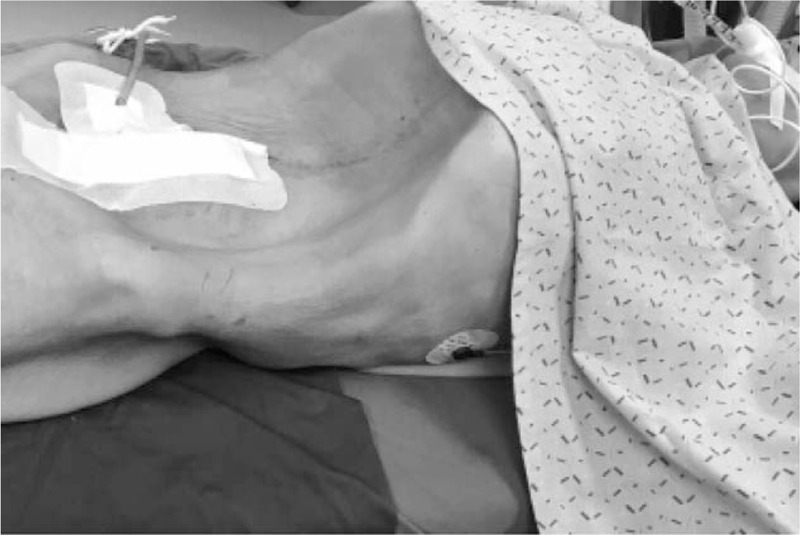
Postoperative appearance of the patient.

During aspiration, although the patient's BP fluctuated greatly, the CVP changed insignificantly from 6 to 8 cmH_2_O. In addition, to prevent postoperative atelectasis, the PEEP was 5 cmH_2_O. The ventilator mode was set to pressure-controlled ventilation, and the same TV was maintained with a lower inspiratory pressure and PEEP to prevent re-expansion pulmonary edema (RPE). During the aspiration process, SpO_2_ was maintained at approximately 100%; draining the tumor fluid improved respiratory compliance, and the patient slowly lifted her head. In the process of removing the ovarian tumor, broad adhesiolysis between the tumor wall and abdominal wall required a long period time and resulted in excessive blood loss. After the tumor was removed, persistent bleeding and low ABP and CVP were observed. After a period of fluid resuscitation and the use of vasoactive drugs, the blood volume was gradually restored, and the HR, arterial pressure and CVP began to return to normal values. To prevent critical intestinal distension and facilitate breathing, an abdominal binder was applied postoperatively, and the patient returned to the intensive care unit (ICU) with an endotracheal tube (Fig. 2). During the operation, the patient's oropharyngeal temperature was in the normal range, and the acid-base balance and electrolyte of the blood gas analysis were normal after transferred to the ICU. Pathologically, the tumor was a mucinous cystadenoma of the left ovary. Intraoperative blood loss was estimated at 1000 mL, 3U RBC, 500 mL fresh frozen plasma, 2000 mL crystal solution, and 500 mL colloidal solution were injected. The duration of surgery was 300 minutes.

Postoperatively, the patient was transferred to the ICU for further monitoring. We used multimodal analgesia after surgery: epidural analgesia assisted nonsteroidal anti-inflammatory analgesics. To avoid RPE, ventilation was provided with an inspiratory pressure of 18 cmH_2_O and PEEP of 8 cmH_2_O, which resulted in a TV of 400 mL. After the patient recovered to spontaneous respiration, she was ventilated with pressure support and PEEP to maintain a TV of 350 to 400 mL. Her respiratory status was good thereafter. The patient was able to generate TVs of 500 mL. The arterial oxygen tension (PaO_2_) was 12 kPa with an inspired oxygen concentration of 30%. The tracheal catheter was removed on postoperative day 2. The postoperative chest X-ray showed no evidence of lung oedema.

After the drainage of large ovarian cysts during surgery, patients are prone to developing RPE postoperative. Therefore, the CVP, arterial pressure, and pulse rate were monitored continuously. Daily estimations of hemoglobin, urea, electrolytes, and serum albumin were made. The patient's basic vital signs remained stable after surgery. In the ICU, we monitored the patient's body temperature, and her armpit temperature was 37.5°C. The arterial blood gas analysis demonstrated normal acid-base balance.

The patient returned to the general ward on postoperative day 3. The epidural catheter was removed before the patient returned to the ward. As patients are at high risk for perioperative thrombosis, low-molecular-weight heparin was administered by subcutaneous injection for symptomatic treatment starting on the third postoperative day.

**Figure 3:**
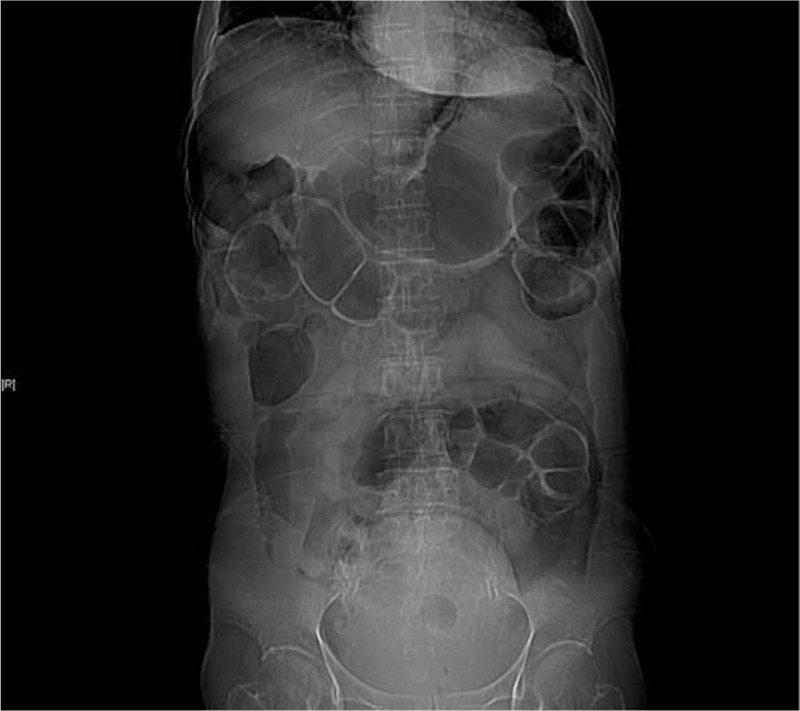
Abdominal CT shows gaseous distention of the intestine.

In addition to low-grade fevers caused by atelectasis, the patient felt considerable abdominal distention on postoperative day 7 and underwent abdominal enhanced CT, which showed a large amount of gas in the abdominal cavity (Fig. 3). As we believed that the patient may have intestinal distension, even though the patient was on a liquid diet, the patient was given a diet restriction. Abdominal distension and abdominal pain were improved by fasting, inserting a nasogastric tube, injecting omeprazole and somatostatin. On postoperative day 10, the patient's abdominal distension significantly improved compared with her previous condition. After a period of observation, the patient was in stable condition, and was discharged 22 days after surgery and was followed up regularly.

## 3 Discussion

Extra-large benign and malignant cysts of the ovary are uncommon and involve diagnostic and management challenges; the determination of CA-125 can help to identify epithelial tumors of the ovary. Almost 90% of ovarian cancers develop from the surface epithelium of the ovaries. As is typical of all primary mucinous tumors, cystadenomas are characteristically unilateral. Only approximately 5% are bilateral. Cystadenomas are generally large tumors that occasionally reach massive proportions.^[1,2]^ Due to the large size of the abdominal tumor, symptoms of acid secretion, bloating, and abdominal compression may occur and may even lead to pleural effusion through the diaphragm.^[3]^

The perioperative management of patients with giant ovarian tumors is associated with several risks and challenges. The following 5 main problems related to surgery were focused on drainage methods; anesthesia; intestinal distention; postoperative analgesia; and a multidisciplinary approach.

### 3.1 Drainage methods

The potential risks of drainage include abdominal fluid leakage and the spread of ovarian cancer, as well as an elevated risk of abdominal infection, bleeding, and adhesions. The researchers advise avoiding drainage from a pathologic perspective. Regarding intraoperative drainage, an increasing number of researchers prefer slow drainage at 0.5–1 L/min.^[4,5]^ In our case, due to the patient's relatively stable condition and the patient's nervous mood, we drained the ovarian cyst safely during surgery.

### 3.2 Anesthesia

Anesthetic management is of vital importance. In particular, the respiratory and cardiovascular management of these patients is critical. The problems involving the circulatory system during the resection of giant ovarian tumors can be divided into the following aspects: before the tumor is removed, supine hypotensive syndrome can be induced by compression of the tumor; a rapid decrease in thoracic pressure and abdominal pressure after the removal of giant ovarian tumors can cause hemodynamic collapse; intraoperative bleeding can occur; and changes in intrathoracic pressure due to postural and intraoperative positive pressure ventilation can also affect hemodynamic factors. Morrison et al found episodes of hypotension during surgery that were not associated with changes in venous pressure and demonstrated the inadequacy of CVP monitoring as a guide to circulating blood volume and ventricular function. The authors emphasized that measuring the pulmonary capillary wedge pressure (PCWP) would have been valuable during the operation.^[6]^ The use of pulmonary catheters, transesophageal Doppler ultrasound, heart function tests, and FloTrac are common in managing the anesthesia in such patients. In recent years, stroke volume variation (SVV) and pulse pressure variation (PPV) have been used to predict fluid responsiveness.^[7]^ In our patient, we used CVP as an indicator of cyclic monitoring due to our limited access to monitoring equipment. We also encountered excessive bleeding during adhesiolysis between the tumor wall and abdominal wall. Patients with giant tumors should undergo careful preoperative evaluations.

Moreover, there was a risk for RPE after the tumor was removed. Pulmonary edema has been reported following surgery to treat a giant intra-abdominal cyst.^[8]^ Pulmonary oedema may occur after the tumor is removed due to the sudden re-expansion of a chronically collapsed lung, which occurred because of compression from the elevated abdomen. RPE is a rare but fatal complication that occurs after rapid re-expansion of a chronically collapsed lung.^[9]^ Implicated in the etiological process of RPE are mechanical features such as duration and severity of collapse, technique of re-expansion, increased pulmonary vascular permeability, airway obstruction, pulmonary artery pressure changes, or inflammatory changes (production of interleukin 8, leukotriene B4, and loss of surfactant) within the pulmonary vasculature.^[10]^ In our case, we successfully prevented intraoperative and postoperative pulmonary edema by applying intraoperative lung protection strategies. To prevent RPE, we chose to re-expand the collapsed lungs very slowly, and we maintained a relatively low TV after the tumor was removed, similar to that during preoperative spontaneous respiration. Using this approach, we were able to manage the patient uneventfully during and after the operation.

### 3.3 Intestinal distention

Postoperative intestinal distention is probably due to a combination of sympathetic activity and diffusion of gases into the bowel lumen following decompression. Intestinal ileus has been reported in several cases. The risk for intestinal distention may be reduced by the use of an abdominal binder and a nasogastric tube.^[8]^ In our case, we were not aware of the dangers before surgery. The patient developed intestinal distention on postoperative day 7. After the nasogastric tube had been placed and gastrointestinal protective drugs had been given, the patient recovered smoothly.

### 3.4 Postoperative analgesia

Severe postoperative pain can be detrimental to the postoperative recovery. Therefore, in this case, postoperative analgesia was considered important, and we decided to use multimodal analgesia after surgery: epidural analgesia assisted by nonsteroidal anti-inflammatory analgesics. In our case, we can see the advantages of multimodal analgesia. Patients can undergo early ambulation and physical therapy to help recover bowel function.

### 3.5 A multidisciplinary approach

A multidisciplinary approach to preoperative planning is critical to achieving intraoperative success and favorable postoperative results in the management of patients with giant abdominal masses. A multidisciplinary approach is important for the resection of large tumors and for a remarkably uneventful postoperative recovery.^[11]^

In conclusion, we have described a procedure for the successful perioperative management of a patient with a giant ovarian tumor. Large ovarian tumors are rarely encountered in modern clinical practice. As many studies have suggested, a multidisciplinary approach is essential. The potential problems associated with the removal of giant ovarian tumor are remarkable, including operative and postoperative complications of cardiovascular dysfunction or ventilatory inadequacy. Furthermore, knowing the possibility of complications and choices for management can lead to better outcomes in such rare cases. Clinical anesthesiology is gradually moving toward perioperative medicine, and the concept of anesthesiology is being updated.

## Author contributions

**Resources:** Junqiao Mi, Yan Jiang.

**Supervision:** Shiduan Wang, Shengnan Cai.

**Writing – original draft:** Junqiao Mi, Rui Dai.

**Writing – review & editing:** Junqiao Mi

## Abbreviations

Abbreviations: ABP = arterial blood pressure, ASA = American Society of Anaesthesiologists, BIS = bispectral index, CT = computerized tomography, CVP = central venous pressure, ICU = intensive care unit, MDT = multidisciplinary teamwork, PCWP = pulmonary capillary wedge pressure, PEEP = positive end-expiratory pressure, PPV = pulse pressure variation, RPE = re-expansion pulmonary edema, SVV = stroke volume variation, TV = tidal volume.
